# Genetic Control of the Variable Innate Immune Response to Asymptomatic Bacteriuria

**DOI:** 10.1371/journal.pone.0028289

**Published:** 2011-11-28

**Authors:** Jenny Grönberg Hernández, Fredrik Sundén, John Connolly, Catharina Svanborg, Björn Wullt

**Affiliations:** 1 Department of Microbiology, Immunology and Glycobiology, Institute of Laboratory Medicine, Lund University, Lund, Sweden; 2 Department of Urology, Skåne University Hospital, Malmö, Sweden; 3 Singapore Immunology Network (SIgN), Biomedical Sciences Institutes, Agency for Science, Technology, and Research (A*STAR), Singapore, Singapore; Columbia University, United States of America

## Abstract

The severity of urinary tract infection (UTI) reflects the quality and magnitude of the host response. While strong local and systemic innate immune activation occurs in patients with acute pyelonephritis, the response to asymptomatic bacteriuria (ABU) is low. The immune response repertoire in ABU has not been characterized, due to the inherent problem to distinguish bacterial differences from host-determined variation. In this study, we investigated the host response to ABU and genetic variants affecting innate immune signaling and UTI susceptibility. Patients were subjected to therapeutic urinary tract inoculation with *E. coli* 83972 to ensure that they were exposed to the same *E. coli* strain. The innate immune response repertoire was characterized in urine samples, collected from each patient before and after inoculation with bacteria or PBS, if during the placebo arm of the study. Long-term *E. coli* 83972 ABU was established in 23 participants, who were followed for up to twelve months and the innate immune response was quantified in 233 urine samples. Neutrophil numbers increased in all but two patients and in an extended urine cytokine/chemokine analysis (31 proteins), the chemoattractants IL-8 and GRO-α, RANTES, Eotaxin-1 and MCP-1, the T cell chemoattractant and antibacterial peptide IP-10, inflammatory regulators IL-1-α and sIL-1RA and the T lymphocyte/dendritic cell product sIL-2Rα were detected and variably increased, compared to sterile samples. IL-6, which is associated with symptomatic UTI, remained low and numerous specific immune mediators were not detected. The patients were also genotyped for UTI-associated *IRF3* and *TLR4* promoter polymorphisms. Patients with ABU associated *TLR4* polymorphisms had low neutrophil numbers, IL-6, IP-10, MCP-1 and sIL-2Rα concentrations. Patients with the ABU-associated *IRF3* genotype had lower neutrophils, IL-6 and MCP-1 responses than the remaining group. The results suggest that the host-specific, low immune response to ABU mainly includes innate immune mediators and that host genetics directly influence the magnitude of this response.

## Introduction

The symptoms and severity of urinary tract infection (UTI) reflect the host response to the infecting strain. In patients with acute pyelonephritis, bacteria trigger a local inflammatory response in the urinary tract, detected as an increase in urine neutrophils and cytokine levels [1], [2], [3]. In addition, the systemic involvement in acute pyelonephritis causes fever and elevated acute phase reactants like C-reactive protein (CRP) [4], [5], [6] and in about 30% of adults, pyelonephritis is accompanied by bacteremia. Patients with asymptomatic bacteriuria (ABU), in contrast, are protected from the development of acute pathology due to a weak host response to infection [7]. They may also be protected from re-infection, if the strain that they carry outcompetes more pathogenic strains [8]. However, variation in the host response has also been noted in patients with ABU, leading to uncertainty about the extent of innate immune reactivity in ABU. Neutrophil numbers in urine vary greatly among patients with ABU and the diagnostic value of pyuria has been debated in this patient group [6]. To use host response parameters as a basis for diagnostic and therapeutic decisions in clinical practice, such variability needs to be assessed [6], [9]. More recent and extensive information on the variable immune repertoire in patients with ABU is lacking, however.

Innate immunity controls the antibacterial defense in the urinary tract and effector molecules include mucosal cytokines, chemokines and antibacterial peptides as well as recruited inflammatory cells [1], [10], [11], [12], [13]. Uroepithelial recognition of *E. coli* triggers the innate immune response and specifically, the Toll-like receptor (TLR) 4 signaling pathway is critically involved [10], [14]. Downstream activation of transcription factors IRF3 or NF-κB stimulates the transcription of chemokine genes and increases e.g. IL-8 and IL-6 expression in the urinary tract [15]. Mice lacking *Tlr4* develop an ABU like state without acute tissue inflammation [10] and in patients with ABU, reduced TLR4 expression has been detected [7]. Mice lacking *Irf3*, in contrast, develop severe acute pyelonephritis with urosepsis and renal damage [15]. Polymorphisms in the *TLR4* and *IRF3* promoters have been detected [16], [17] and associated to ABU [15], suggesting that the genetic repertoire of the host contributes to the reduced innate immune response in this patient group. Recent genetic screens in women with and without ABU detected an association between polymorphisms in *TLR2* and *CXCR1*, but their effect on the cellular immune response repertoire in ABU and the effects of bacterial strain diversity have not been considered [18].

Further complicating the understanding of the host response to ABU is the origin of the infecting strain and resulting variability in virulence factor expression [19], [20], [21], [22]. To specifically evaluate the host response to ABU, we have examined samples from patients subjected to therapeutic inoculation with the ABU prototype strain *Escherichia coli* 83972 [8]. Through this unique approach, we have excluded the bacterial strain variation accompanying natural infection. The host response was characterized by broad urine proteomic profiling, and the host *TLR4* and *IRF3* genotypes were determined. The results show that ABU elicits a low, host specific innate immune response and that the patient genotype influences the propensity to mount a host response to ABU.

## Results

### Patients and samples

**Figure 1:**
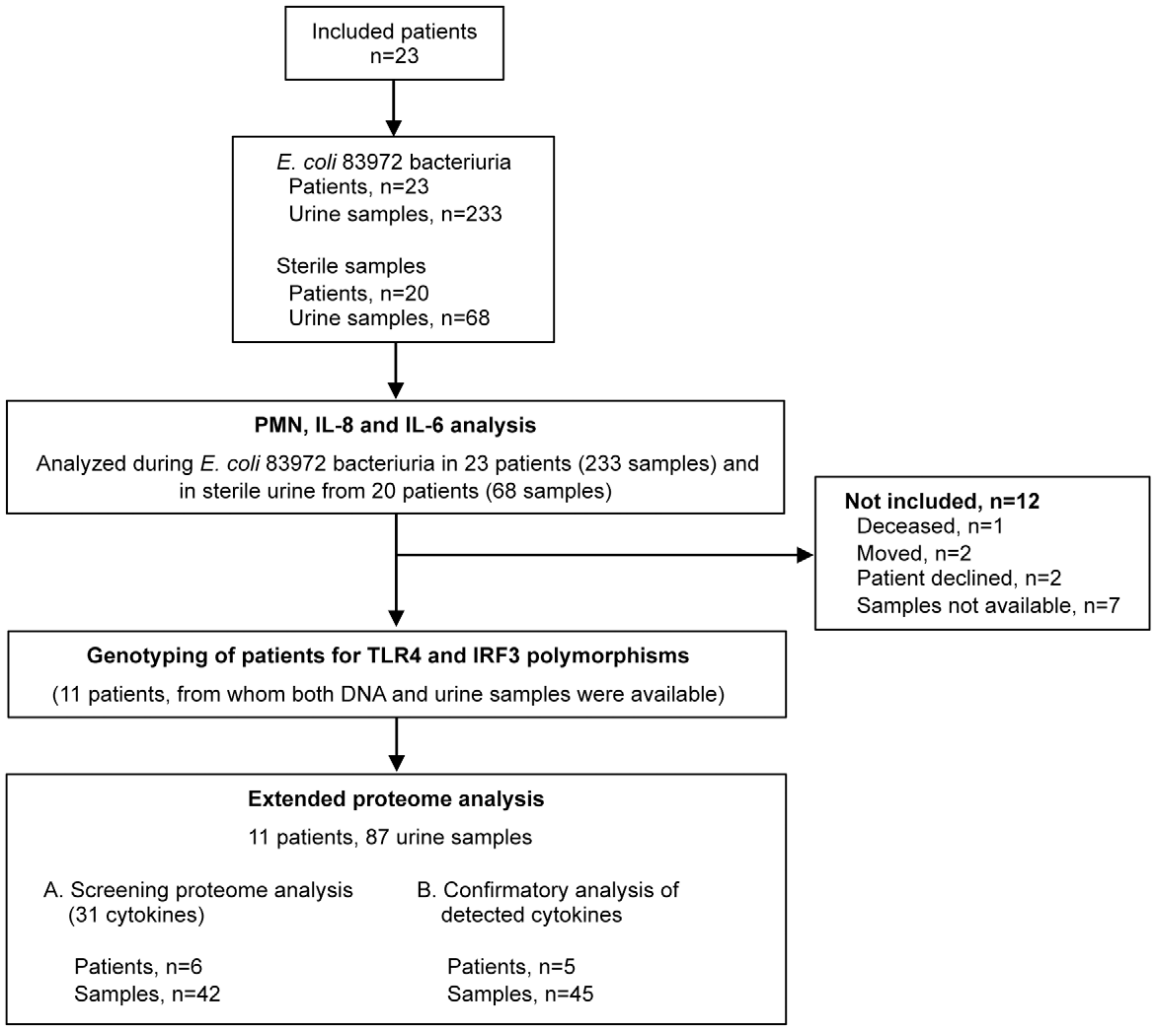
Patients and samples. Samples from patients participating in a clinical trial of induced *E. coli* 83972 ABU were analyzed. All collected urine samples were subjected to PMN, IL-6 and IL-8 quantification, and blood samples from eleven patients were collected for genotyping of promoter polymorphisms in TLR4 and IRF3. Blood and urine samples from these eleven patients were also selected for an extended urine protein analysis.

To characterize the host response to ABU, we included 23 patients participating in a placebo-controlled study of therapeutic *E. coli* 83972-inoculation (Table 1) [8]. In patients who developed *E. coli* 83972 ABU, 233 *E. coli* 83972-positive urine samples representing 260 months of observation were collected (mean 11.4, range 3.8–19.1 months). Sterile samples collected from the same patient group (20/23 patients) after saline inoculations in the placebo arm of the study, were used to distinguish the host response to *E. coli* 83972 from protein secretion into sterile urine (Figure 1).

**Table 1:** Patient Characteristics.

			*E.coli* 83972 ABU			Sterile	
Patient ID^1^	Age	CIC^2^	Inoculation attempts	Months	Urine samples	Urine samples	Diagnosis
*lower motor neuron lesions, residual urine*							
M1	77	+	2	9.4	11	6	Idiopathic detrusor insufficiency
F1	59	+	2	11.8	12	1	Idiopathic detrusor insufficiency
F2	66	+	1	12.2	11	3	Residual urine after urethropexia
F3	46	0	1	12.1	13	3	Detrusor insufficiency; after borreliainfection
F4	84	0	1	10.5	10	0	Idiopathic detrusor insufficiency
F5	82	+	1	12.0	10	2	Idiopathic detrusor insufficiency
F6	77	0	1	8.3	9	5	Idiopathic detrusor insufficiency; coronary by pass surgery
F7	45	0	3	5.2	4	3	Detrusor insufficiency; diabetes mellitus type 1
F8	76	0	2	3.8	4	2	Idiopathic detrusor insufficiency
F9	64	0	1	12.0	12	4	Residual urine after urethropexia; cystocele
M2	72	+	1	11.6	16	5	Detrusor insufficiency; after encephalitis
F10	32	0	1	10.7	9	5	Idiopathic detrusor insufficiency
*spinal lesions*							
M3	76	+	2	19.1	10	1	Tetraplegia
M4	39	0	1	11.1	10	6	Tetraplegia; spinal cord injury
M5	47	+	3	17.3	10	0	Tetraplegia; spinal cord injury, diabetes mellitus type 2, sphincterotomy.
F11	60	+	3	10.4	9	4	Tetraplegia; epidural hematoma
M6	52	+	1	16.8	14	5	Tetraplegia
F12	61	+	1	10.8	11	0	Paraplegia; slipped disc, epidural hematoma
M7	51	+	1	4.0	3	2	Tetraplegia; spinal cord injury
M8	68	+	2	16.6	11	1	Tetraplegia
M9	38	+	1	12.2	13	4	Tetraplegia; spinal cord injury
M10	55	+	1	12.2	12	3	Tetraplegia; epidural hematoma
M11	45	+	1	11.0	9	3	Paraplegia; spinal cord injury

The table displays data from trials performed between 1993 and June, 2005. All patients had incomplete bladder emptying (residual urine ≥100 ml) and UTI susceptibility with a history ≥3 UTI/ year with urinary cultures showing uropathogenic growth, two years prior to the study.

1) M =  Male, F =  Female.

2) Clean Intermittent Catheterization. All patients had been instructed to use CIC regularly. Of the 8 patients who did not use CIC during the study 2 patients refused because of practical reasons and the remaining 6 patients had residual urine <300 ml, and had not experienced any improvement from previously performed regular CIC.

### Basic host response parameters; PMN, IL-8 and IL-6 responses to E. coli 83972 ABU

**Figure 2:**
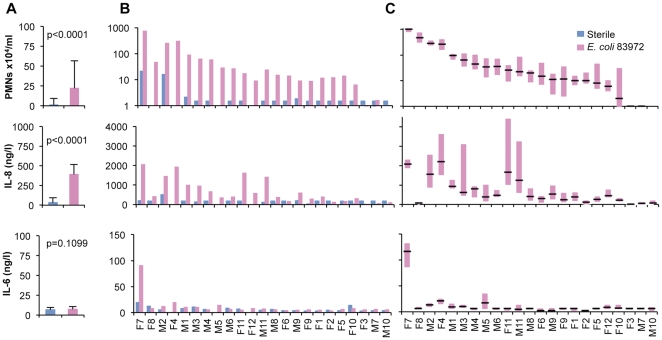
Host response to *E. coli* 83972 bacteriuria. *E. coli* 83972 ABU triggered an increase in PMN numbers and IL-8 concentrations (p<0.0001) but IL-6 levels were unchanged (n.s., Mann-Whitney test). Group-wise comparison of monthly urine samples collected during *E. coli* 83927 ABU or after PBS inoculations. Coded patient IDs are noted on the x-axis. A. Means + SEs of neutrophil numbers, IL-8 and IL-6 concentrations during *E. coli* 83972 bacteriuria (pink) or sterile conditions (blue). B. Intra-individual comparison of samples obtained during *E. coli* 83972 bacteriuria (pink) and sterile intervals (blue). C. Box-plot of intra-individual host response variation during *E. coli* 83972 ABU.

All urine samples were initially screened for neutrophil numbers and IL-6/IL-8 concentrations. Significant PMN and IL-8 responses to ABU (both p<0.0001) were detected compared to sterile samples (p<0.0001, Figure 2A) with elevated levels in 21/23 patients (Figure 2B). Intra-individual variability during long-term *E. coli* 83972 ABU was also evaluated in repeat samples from each patient (Figure 2C). The host response variation was more limited for neutrophil numbers than for IL-8 concentrations. Variability in neutrophil counts was lower in high responders (median >100 x neutrophils/ml, n = 4), but the opposite was observed for the IL-8 concentrations, which were more stable in the low responders (median <500 ng/l, n = 14) (Figure 2C). No IL-6 response was detected (Figures 2A–C).

**Figure 3:**
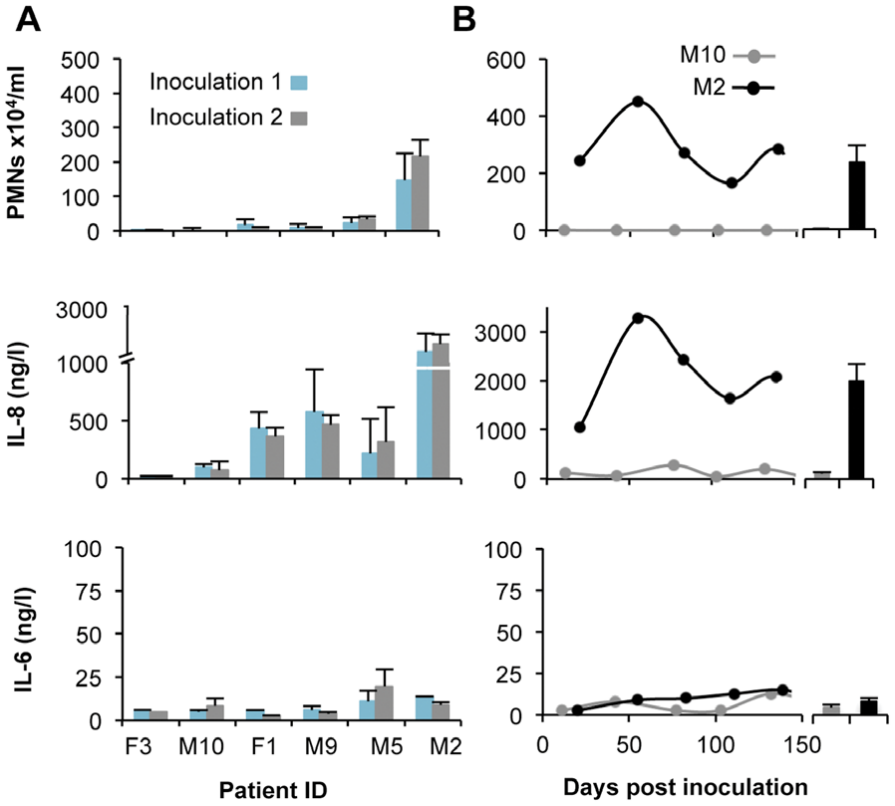
Consistency of the individual host response to *E. coli* 83972 inoculation. A. The host response in urine samples from the first (blue) and second inoculations (grey) were compared (Geometric means + SEs) in six patients that had received repeated inoculations. B. Kinetics of the host response during the first and second ABU episode in one high and one low responder.

To further address if the magnitude of the host response to *E. coli* 83972 ABU was stable in each individual, PMN, IL-8 and IL-6 responses after the first and second inoculations were compared in the six patients who were colonized with *E. coli* 83972 twice and developed bacteriuria periods lasting at least three months (total n = 75 urine samples). Neutrophil and IL-8 responses to repeat inoculations were highly reproducible (Figure 3A). The kinetics of PMN, IL-8 and IL-6 responses during the first 150 days after inoculation are shown in Figure 3B for one high and one low responder. The results suggest that ABU elicits a variable innate host response, the level of which is host specific.

### Urine cytokine/chemokine proteome during E. coli 83972 ABU

**Figure 4:**
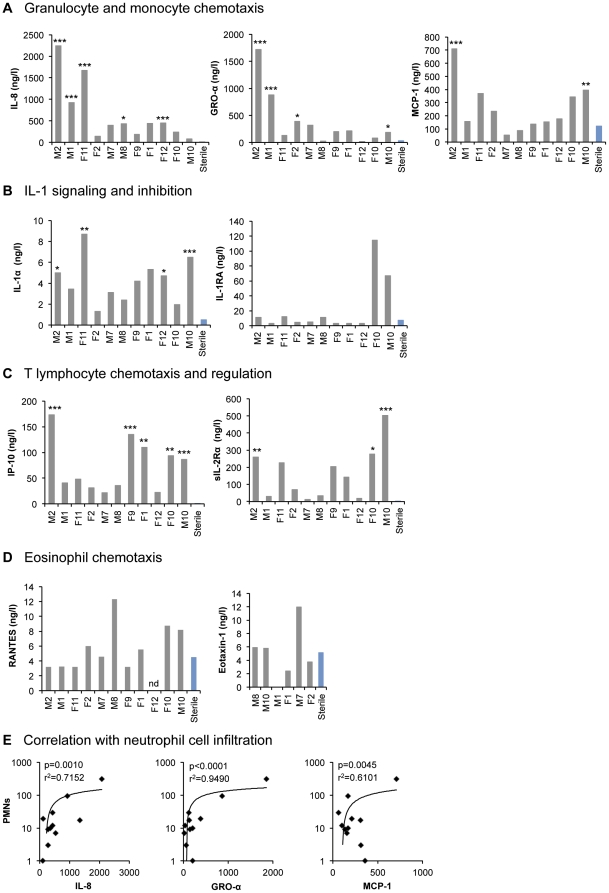
Urine cytokine/chemokine proteome response to *E. coli* 83972 ABU. Cytokine/chemokine levels in individual patients during ABU (grey, medians of all samples in each individual). A pool of 20 sterile urine samples from 9 patients (blue, median of all samples) is used for comparison. Coded patient IDs are noted on the x-axis, in the same order as in Figure 1. Significant differences between sterile and ABU samples from individual patients (Kruskal-Wallis, Dunn's post test) are shown (*p<0.05, **<0.01, ***<0.001). A. Granulocyte (IL-8, GRO-α) or monocyte (MCP-1) chemotaxis. B. Cytokines involved in IL-1 signaling and inhibition. C. Cytokines involved in T lymphocyte chemotaxis and regulation. D. Cytokines involved in eosinophil chemotaxis. E. Scatter diagrams illustrate significant correlations between neutrophil counts and neutrophil chemoattractants IL-8 and GRO-α, and the monocyte chemoattractant MCP-1. For the remaining proteins, no correlation with neutrophil numbers was found. For sample numbers, group means and statistical analysis see Table 2.

Human uroepithelial cells respond to infection by secreting cytokines and chemokines. The response is further modified by the activation of other resident and recruited cells, including neutrophils that predominate in the acute cellular infiltrate during UTI [11]. To further characterize the mucosal host response repertoire in patients with *E. coli* 83972 ABU, 87 urine samples from eleven patients were subjected to extended urine cytokine/chemokine proteome analysis (Table 2 and Figure 4). Six patients (42 urine samples) were selected for an extensive urine cytokine/chemokine screening of 31 immune-related protein proteins. The IL-8 and IL-6 concentrations obtained in the screen corresponded to the results previously obtained by the Immulite method. In addition, the screen revealed the presence in urine of seven immune mediators, GRO-α, IP-10, MCP-1, sIL-2Rα, IL-1α, sIL-1RA and RANTES. Nineteen proteins were not detected and three were detected in four (Eotaxin-1, 32 samples), three (IL-12p40, 14 samples) and one patient (IFNα_2_, one sample, see Table 2). The detected proteins were subsequently quantified in the remaining urine samples, using a custom made assay (five patients, 45 samples).

**Table 2:** Groupwise analysis of the urine cytokine/chemokine proteome response to *E. coli* 83972 ABU.

	*E. coli* 83972 ABU			Sterile^c^			
Host response Parameter^a^	Mean^b^		SEM	Mean		SEM	p-value^d^
PMNs	46.0	±	26.8	2.1	±	1.2	0.0038
IL-8	654.8	±	209.3	50.2	±	35.9	0.0004
IL-6	5.5	±	1.0	3.2	±	0.5	0.2984
GRO-α	379.2	±	152.1	32.3	±	9.1	0.0062
IP-10	72.6	±	15.4	0.1	±	11.2	0.0138
MCP-1	265.5	±	56.8	121.6	±	22.0	0.0583
IL-1α	4.2	±	0.6	0.5	±	0.3	0.0024
IL-1RA	21.6	±	10.8	7.3	±	3.9	0.4466
sIL-2Rα	161.5	±	45.8	0.1	±	34.0	0.2537
RANTES	5.82	±	0.97	4.51	±	6.9	0.1733
Eotaxin-1	4.8	±	1.8	5.1	±	1.7	
IL-12p40	15.7	±	3.0	-			
IFN-α_2_	20.5	±	-	-			

a Parameters were analyzed in 87 samples from 11 patients, except for RANTES which was analyzed in 64 samples from ten patients, and Eotaxin-1, IL-12p40 and IFN- α2 in 42 samples from six patients. Not detected: CCL22, MIP-1α, GM-SCF, G-CSF, MCP-3, sFasL, sICAM-1, IL-1β, IL-2, IL-3, IL-4, IL-5, IL-7, IL-10, IL-12p70, IL-13, IL-15, TNF-α, IFN-γ.

b Mean of individual median values for each protein from eleven patients.

c Mean of individual median values for each protein from nine patients, except for Eotaxin-1 (mean from 6 patients).

d Mann-Whitney test.

In addition to IL-8 (Table 2, p<0.0004), the cytokine array detected significantly increased levels of the neutrophil chemoattractant GRO-α during ABU in the entire patient group compared to all sterile controls (Table 2, p<0.0062) and by intra-individual analysis significantly increased levels were detected in four individuals. A significant correlation between IL-8, GRO-α concentrations and neutrophil numbers was detected in individual patients (p = 0.001 and, p<0.0001 respectively, Figure 4E). By group-wise analysis, no significant MCP-1 response was detected but median levels in 9/11 patients were higher than in sterile urine and in two patients, a significant increase was observed compared to the sterile samples (Figure 4A). A correlation of the monocyte chemoattractant MCP-1 with neutrophils was also detected (p = 0.0045, Figure 4E) but was limited to high neutrophil numbers, when the more complex inflammatory infiltrate might include monocytes.

Uroepithelial cells express IL-1 in two forms; IL-1α, which is mainly membrane-bound and IL-1β, which is a secreted cytokine [23], [24]. A significant IL-1α response to ABU was detected in the entire patient group (p = 0.002, Figure 4B), with elevated median concentrations in all patients and in four patients a significant increase was observed compared to the individual sterile samples. Increased median concentrations of the IL-1 receptor inhibitor soluble IL-1RA were present in 5/11 patients (n.s.), but the levels did not correlate with IL-1α concentrations (data not shown). The IL-2 decoy receptor, soluble IL-2Rα was detected in all patients and increased significantly after *E. coli* 83972 inoculation in three patients. Interestingly, the highest sIL-1RA and sIL-2Rα responses occurred in the same two patients.

The monocyte/T lymphocyte chemoattractant IP-10, which also has antibacterial activity [25], was significantly increased by ABU in the entire patient group (p<0.02, Table 2). Median levels of IP-10 during ABU were elevated in all patients compared to sterile urine, and significantly elevated in five patients compared to the sterile samples (Figure 4C). Low levels of the eosinophil and T cell chemoattractant RANTES were detected in all patients, as compared with sterile samples (Figure 4D). No significant RANTES response to *E. coli* 83972 ABU was observed, however (n.s., Table 2 and Figure 4D). Eotaxin-1, IFNα_2_ and IL-12p40 were detected in a limited numbers of samples (Figure 4D and Table 2).

### Genetic polymorphism influence the host response

**Figure 5:**
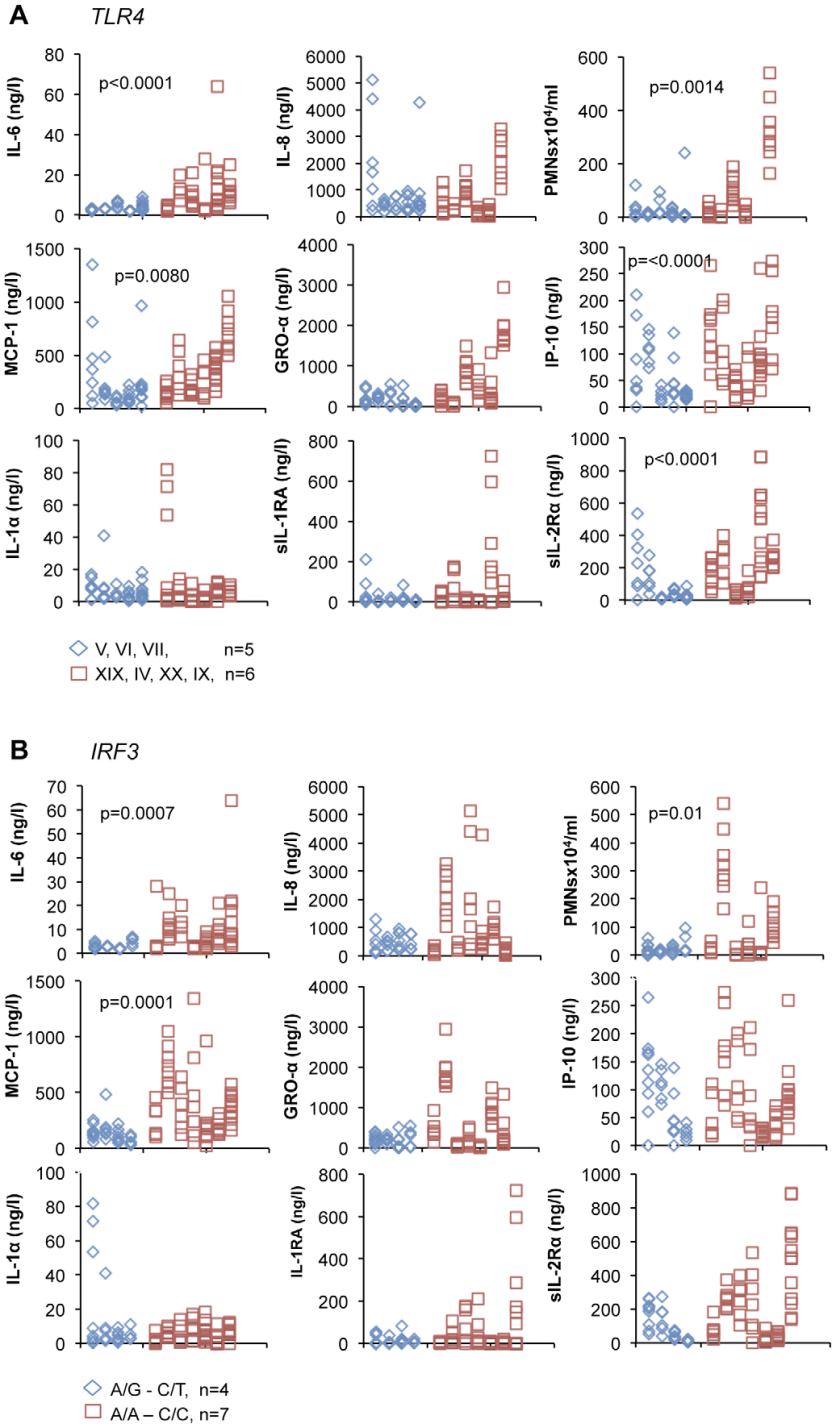
Promoter polymorphisms and the host response to ABU. A. Three of five *TLR4* genotypes associated with primary ABU were detected (blue hexagons) in five of the eleven patients. These patients had significantly lower neutrophil numbers (p<0.002) and IL-6 (p<0.0001), MCP-1 (p<0.01), IP-10 (p<0.0001), and sIL-2Rα (p<0.0001) concentrations than the patients with non-ABU associated *TLR4* genotypes XIX, IV, XX and IX (red squares). Each column represents one patient and each hexagon or square one monthly urine sample. B. The heterozygous *IRF3* promoter genotype associated with ABU (A/G-C/T, blue hexagon) was detected in four of the eleven patients, who had significantly lower neutrophil numbers (p = 0.01) and IL-6 (p<0.001) and MCP-1 (p = 0.0001) concentrations than patients with the homozygous, pyelonephritis-associated genotype (A/A-C/C, red square). Each column represents one patient and each hexagon or square one monthly urine sample.

To investigate if genetics influence the host response to ABU, *TLR4* and *IRF3* promoter polymorphisms were defined in the eleven subjects, whose urine was subjected to cytokine/chemokine proteome analysis. Seven of the 29 *TLR4* genotypes that occur in the Swedish population [16] were identified. The five subjects who carried *TLR4* genotypes associated with primary ABU (V, VI or VII) [16], had significantly lower IL-6, MCP-1, IP-10, sIL-2Rα and PMN responses to ABU than patients with other *TLR4* genotypes (XIX, IV, XX, IX, Figure 5A). In addition, four patients carried the heterozygous *IRF3* promoter variant (A/G – C/T) that is more common in ABU than in APN patients [15]. These patients had significantly lower urine concentrations of IL-6, MCP-1 and lower PMN numbers than homozygous patients (Figure 5B). The results suggest that genetic variation influences the innate immune response to ABU.

## Discussion

ABU represents one extreme of the UTI spectrum and may even be regarded as a form of commensalism [12]. Epidemiologic studies have convincingly shown that in most cases, ABU is harmless and the demonstration that bacteriuria protects against super-infection with more virulent strains has provided the rationale for therapeutic establishment of ABU [5], [11], [18]. While it is now widely accepted that ABU is beneficial and should be left untreated, there have long been concerns that some patients with the appearance of ABU may have chronic renal infections, which should be treated to avoid tissue damage [26]. Distinguishing ABU from other, therapy-requiring forms of UTI therefore remains a diagnostic challenge. Refined molecular mapping of the host response repertoire is now possible, offering a promising tool to help resolve diagnostic enigmas in the future, to reduce the risk for tissue damage and the need for antibiotic treatment [27].

The mucosal cytokine response to infection was discovered in UTI and epithelial cells were identified as the first line of cytokine responder cells [28], [29]. Since then, numerous studies have examined the cytokine response magnitude and repertoire in patients with different forms of UTI [30]. Early work showed that acute pyelonephritis is accompanied by an increase in urine IL-6 compared to ABU [3], [31] and IL-8 levels showed a similar pattern [1], [32], [33], defining ABU as a condition with low mucosal host response induction. Increased serum levels of IL-6 and IL-8 were detected in bacteremic patients [31], [34], [35] and further work investigating the cytokine repertoire showed that febrile UTI causes an increase in GRO-α and ENA-78 in urine, and ENA-78 in serum [35]. Acute cystitis has been associated with an increase in urine IL-1α, GRO-α, IL-1β, IL-6, IL-8 and TNF-α compared to sterile samples [36], [37], [38]. Urine cytokine levels in ABU have been examined in transplant patients [39] and diabetic children with ABU [40]. A broader urine cytokine repertoire was examined in elderly subjects, where a group wise comparison of samples from ABU patients and sterile controls, detected a significant increase in TNF-α, IL-12, IL-18, GRO-α, IL-8, IL-6 and IL-10 in the ABU group, while MCP-1 and IL-1β were detected but not increased [38]. A comparison with acute cystitis samples showed that the levels of IL-6 and IL-8 were lower in ABU. All ABU patients in the study carried *E. coli* but the properties of the infecting strains were not recorded and host genetics were not considered.

In the present study, the host response was characterized in patients, who developed bacteriuria with the prototype ABU strain *E. coli* 83972 after therapeutic inoculation. By selecting this patient group, the study focused on host-driven differences in the innate immune response to a single ABU strain and bacterial differences accompanying natural infection were minimized. By extending the cytokine proteomic analysis to include 31 immune response related proteins, several interesting host response features were observed. *E. coli* 83972 bacteriuria stimulated a neutrophil response and the concentrations of IL-8 and GRO-α correlated well with the level of pyuria in individual samples. These neutrophil chemoattractants [1], [41] are important for the recruitment neutrophils and activate their antibacterial effector functions, which are crucial to eradicate bacteria during acute UTI [42]. The monocyte chemoattractant MCP-1 showed variable levels, which are difficult to interpret, as there is little information on monocyte infiltration in response to UTI. IL-1α was increased in all patients with *E. coli* 83972 ABU. In previous studies, an IL-1α response was detected in patients with acute cystitis and acute pyelonephritis [36], [37], [43]. A decrease of sIL-1RA during symptomatic UTI has been recorded, suggesting that local inflammation might consume sIL-1RA [43]. As previously noted [43], IL-1α levels did not correlate with those of the inhibitor sIL-1RA. While all patients showed detectable levels of RANTES in the urine samples, there was no increase after bacterial inoculation, suggesting a basic level of activation and cytokine secretion by the mast cells in the bladder mucosa. In a parallel study, we observed a RANTES response in patients who develop acute symptoms, however, reflecting eosinophil activation and a potential role for eosinophil mediators in the development of cystitis (data not shown).

Interestingly, the response to ABU included the T cell chemoattractant IP-10, which has been found to have antibacterial activity against *E. coli* [25]. The secretion of the IL-2 decoy receptor sIL-2Rα in certain patients during both ABU and infection-free intervals may also reflect T cell- or dendritic cell activation in the urinary tract mucosa [44]. The presence of such cells has not been extensively characterized, however it is known that Peyer's patch-like cellular aggregates are formed in the bladder wall of patients with long-term ABU [45]. It may be speculated that such aggregates may include lymphocytes and dendritic cells and constitute a possible source of sIL-2Rα in patients with long-term bacteriuria, as those in the present study as well as during infection-free intervals until involution of these cellular aggregates has occurred. Most of the mediators of T cell proliferation and differentiation were not detected in patient urine, however, including IL-2, IL-3, IL-4, IL-7, IL-12p70. We did not detect T cell chemoattractants (MCP-3), B cell inhibitory cytokines (IL-14), agonists (IL-10) or regulators (IL-13), or agonists of NK cell proliferation (IL-15). Macrophage secreted proteins like the granulocyte activator MIP-1α and CCL22 were also not detected. Their absence supports the established view that adaptive immunity plays a less prominent role in UTI than innate immunity [12].

The TLR4 and IRF3 polymorphisms examined in this study were originally linked to ABU when they were shown to differ between populations who developed symptomatic infections and ABU [15], [16]. The highly selected, UTI prone study populations were genotyped when their infection pattern had been established after many years of follow up. Patients with no evidence of symptomatic UTI during several years of follow up were assigned to a “primary ABU group”. Children who developed ABU after a prior symptomatic infection but with no further symptomatic episodes were assigned to a “secondary ABU” group and the APN group had a history of APN, but had not developed ABU at any time during follow-up [16]. In a subsequent study, adults with a history of childhood APN were reexamined after 30 year follow up and assigned to APN or secondary ABU groups, depending on their disease patterns. Pediatric age-matched controls were enrolled at the pediatric outpatient clinic or when admitted for elective surgery for diagnoses unrelated to infection [46]. The ABU associated TLR4 genotypes used in this study were derived from the “primary ABU” group.

*TLR4* promoter polymorphisms that lowered TLR4 promoter activity were associated with ABU, suggesting that genetic variation affecting TLR4 expression influences UTI susceptibility [16]. We now add data on patients with compromised urinary tracts, showing that individual patients with the ABU associated *TLR4* genotypes have reduced cytokine/chemokine responses to *E. coli* 83972, compared to patients with other *TLR4* genotypes. The transcription factor IRF3, downstream of TLR4 has also been shown to control UTI susceptibility, by affecting the efficiency of the TLR4 pathway further downstream [15]. Gene deletions result in an exaggerated, dysfunctional inflammatory response and in aggravated acute pyelonephritis in *Irf3* ^-/-^ mice. In patients with UTI, polymorphisms affecting the *IRF3* promoter have been detected, and the genotype associated with low promoter function is associated with acute pyelonephritis [15]. In this study, we detected an association between the *IRF3* promoter genotype and high or low responses to *E. coli* 83972, further emphasizing the importance of host genetics for the immune response in the urinary tract. In view of the fact that all patients carried the same *E. coli* strain these findings make an even stronger argument for genetic control of the host response to UTI.

The severity of UTI is explained by the virulence of the infecting strain and by the resistance to infection of individual hosts [12]. Despite the advanced understanding of molecular disease mechanisms, common diagnostic procedures do not routinely include the assessment of these parameters. The traditional clinical assessment of disease severity is usually made without the support of quantitative molecular data and current diagnostic practices create a grey-zone, recognized as a cause of antibiotic over-use, and the need for improved diagnostic markers is obvious [27]. This study illustrates how an assessment of the immune response provides a powerful new tool to quantify and analyze the host response to UTI. In this case, we evaluated if patients with ABU show a local immune response to infection of characterized the repertoire of cytokines/proteins in urine. The results suggest that immune response measurements would add both qualitative and quantitative information. Supporting a positive urine culture with a urine cytokine analysis would be of special value in children, who cannot express their symptoms, in elderly, sometimes cognitively impaired patients with multiple diseases, in patients with neurogenic bladders and detrusor over-activity, in patients treated in the emergency department, and in other patients where UTI/ABU cannot be distinguished by subjective symptoms only. Distinguishing the basic immune response in the urinary tract from responses to pathogenic bacteria during symptomatic infection may also help to distinguish ABU from acute cystitis and to avoid excessive use of antibiotics in patients with compromised urinary tracts.

## Materials and Methods

### Ethics statement

The study was approved by the human ethics committee at Lund University and patients gave their informed consent (Clinical Trial Registration RTP-A2003 (International Committee of Medical Journal Editors, www.clinicaltrials.gov)).

### Study design

Patients with incomplete bladder emptying and recurrent UTI (3-4 UTI/year for 2 years) resistant to conventional therapy participated in a placebo-controlled study of therapeutic *E. coli* 83972 inoculation [8]. The aim of the study was to establish if the deliberate establishment of asymptomatic bacteriuria with *Escherichia coli* 83972 in patients with incomplete bladder emptying and recurrent urinary tract infection protects against recurrent UTI. Exclusion criteria were upper tract UTI, renal deterioration, hydronephrosis, untreated bladder outflow obstruction, urinary calculi, immunosuppression (including corticosteroid medication) or urological malignancies. Before inclusion patients underwent renal function tests, upper urinary tract imaging, urodynamic assessment and cystoscopy. A flow-chart of patients and samples is given in Figure 1.

During phase 1 of the study the patients were randomized to blinded inoculations with *E. coli* 83972 or saline, and crossover occurred after monitoring for 12 months or after a UTI episode. During phase 2 patients were subjected to additional blinded inoculations to extend periods with and without *E. coli* 83972 bacteriuria. Outcomes were 1) the time to the first urinary tract infection in patients with and without *E. coli* 83972 bacteriuria and 2) the number of UTI episodes during 12 months with and 12 months without *E. coli* 83972 bacteriuria. There was no febrile urinary tract infection episode in either of the study arms and no significant side effects of intravesical bacterial inoculation were reported. The study concluded that deliberately induced *E. coli* 83972 bacteriuria protects patients with incomplete bladder emptying, who are prone to urinary tract infection, from recurrent urinary tract infection. The present study identified all urine samples from the patients during asymptomatic periods in the active (n = 233) and in the placebo-arm (n =  68) (Fig. 1, Table 1).

### Inoculation protocol

Briefly, patients were catheterized and inoculated with *E. coli* 83972 (10^5^ cfu/ml in PBS, 30 ml) or placebo (sterile PBS) once daily on three consecutive days. Each patient was followed for twelve months after *E. coli* 83972 inoculation, and twelve months after PBS inoculation. In case bacteriuria was not established, the patient received a new inoculation of *E. coli* 83972. The clinical trial was double-blinded and randomized, [8] with monthly urine sampling after both *E. coli* 83972 and PBS inoculation to determine the establishment of long-term *E. coli* 83972 bacteriuria and the host response.

### Urine sampling and culture

Urine samples were obtained before inoculation and during *E. coli* 83972 ABU (233 samples). 68 samples were obtained during sterile intervals after PBS inoculation. All samples were semi-quantitatively cultured. *E. coli* 83972 was identified by antibiotic resistance and a 1.565 bp plasmid was amplified by PCR. Urine samples were stored at −80°C.

### Host response to E. coli 83972

In all samples, urine polymorphonuclear cell (neutrophil) numbers were quantified by hemocytometry, and Interleukin (IL) 6 and IL-8 concentrations were quantified by Immulite (Siemens, Deerfield, USA). From these samples, urine samples from eleven patients (five males and six females) were selected for further analysis of proteomic content. Sterile samples (total n = 20, from 9 patients) and samples from periods of *E. coli* 83972 ABU (n = 67) from each of the patients were chosen as controls. Samples from six patients were selected for extended proteomic analysis of infected (*E. coli* 83972 ABU) and sterile urine (total samples, n = 42). MILLIPLEX MAP Human Cytokine/Chemokine Panel (Millipore, Billarica, USA) was used to screen for protein content, and a custom-made panel detecting GRO-α, IP-10, MCP-1, sIL-2Rα, IL-1α, sIL-1RA and RANTES was used to confirm the analysis in the remaining five 45 samples (five patients).

### TLR4/IRF3 genotyping

DNA was extracted from heparinized peripheral blood using QIAmp DNA Blood Midi Kit from 11 patients (see Figure 1 for details). Patients were genotyped using a Pyrosequencer (PSQ 96, Biotage, Uppsala, Sweden) after PCR amplification of patient DNA and a second biotinylated PCR for each SNP (for primers see [15], [16]).

### Statistics

Cytokine concentrations were evaluated using the Mann-Whitney test or Paired t-test, except for the SNP-analyses where Pearson's chi-square *χ* ^2^ test was used and for individual comparisons, Kruskal-Wallis with Dunn's post test was used. Correlations were by Spearmans rank test. The GraphPad Prism software for Mac, version 5.0, GraphPad Software was used for all calculations.
