# Correction: Genetic Control of the Variable Innate Immune Response to Asymptomatic Bacteriuria

**DOI:** 10.1371/annotation/ab823a8e-01b1-48b8-8b2a-0993d805fc50

**Published:** 2012-01-11

**Authors:** Jenny Grönberg Hernández, Fredrik Sundén, John Connolly, Catharina Svanborg, Björn Wullt

There is a typographical error in the first author's name. The correct spelling is: Jenny Grönberg-Hernández. The correct citation is: Grönberg-Hernández J, Sundén F, Connolly J, Svanborg C, Wullt B (2011) Genetic Control of the Variable Innate Immune Response to Asymptomatic Bacteriuria. PLoS ONE 6(11): e28289. doi:10.1371/journal.pone.0028289 

